# Tuberculosis Under Biotherapy in Patients with Spondyloarthritis: Data from the Moroccan Biotherapy Registry (RBSMR) during 3 Years of Follow Up

**DOI:** 10.31138/mjr.210324.tub

**Published:** 2025-01-03

**Authors:** Salma Zemrani, Bouchra Amine, Imane Elbinoune, Chaimae Charoui, Samira Rostom, Ihsane Hmamouchi, Redouane Abouqal, Ahmed Bezza, Fadoua Allali, Imane El Bouchti, Abdellah El Maghraoui, Imad Ghozlani, Hasna Hassikou, Taoufik Harzy, Linda Ichchou, Ouafae Mkinsi, Redouane Niamane, Rachid Bahiri

**Affiliations:** 1 Department of Rheumatology A, El Ayachi Hospital, Ibn Sina University Hospital, Salé, Morocco; 2 Faculty of Medicine, Health Sciences Research Center (CReSS), International University of Rabat (UIR), Rabat, Morocco; 3 Laboratory of Biostatistics, Clinical Research and Epidemiology, Mohammed V University, Rabat, Morocco; 4 Department of Rheumatology, Military Hospital Mohammed V, Ibn Sina University Hospital, Rabat, Morocco; 5 Department of Rheumatology B, El Ayachi Hospital, Ibn Sina University Hospital, Salé, Morocco; 6 Department of Rheumatology, Arrazi University Hospital, Marrakech, Morocco; 7 Private Medical Office, Rabat, Morocco; 8 Department of Rheumatology, University Hospital of Agadir, Agadir, Morocco; 9 Department of Rheumatology, Military Hospital Moulay Ismail, Hassan II University Hospital, Meknès, Morocco; 10 Department of Rheumatology, Hassan II University Hospital, Fès, Morocco; 11 Department of Rheumatology, Mohammed VI University Hospital, Oujda, Morocco; 12 Department of Rheumatology, Ibn Rochd University Hospital, Casablanca, Morocco; 13 Department of Rheumatology, Military Hospital Avicenne, Mohammed VI University Hospital, Marrakech, Morocco

**Keywords:** spondyloarthritis, tuberculosis, biotherapy, TNF-inhibitors

## Abstract

**Objective:**

Biologics agents may lead to a significant risk of infection, including tuberculosis, particularly in endemic countries. This study aims to determine the incidence and characteristics of active tuberculosis in spondyloarthritis patients undergoing biotherapies and estimate the rate of reactivation of latent tuberculosis infection (LTBI).

**Methods:**

A prospective multicentre study was conducted based on 3-year data from the Moroccan Register of Biotherapies (RBSMR). We determined the incidence rate of tuberculosis during follow-up and performed a comparison with patients in whom tuberculosis was not detected. Screening for LTBI prior to the initiation of biotherapy was analysed, and the reactivation rate was determined at the 3-year follow-up.

**Results:**

194 patients with SpA were included. 98.8% of the patients received TNF-inhibitors, and 6.6% had a history of treated tuberculosis infection. After 3 years of follow-up, 10 cases of active tuberculosis were recorded with an incidence of 17/1000 patient-years. All of these patients were on TNF-inhibitors. diabetes was significantly higher in patients with active tuberculosis (P=0.02), as was the prior use of at least two TNF-inhibitors (P=0.03). Before initiating biotherapy, 22.6% of individuals were found to have LTBI and received chemoprophylaxis. After a 3-year follow-up, only 2 (4.5%) cases of active TB were noted in patients previously treated for LTBI whereas the other 8 cases had negative screening.

**Conclusion:**

This study suggests that patients undergoing biotherapy, particularly TNF-inhibitors have a higher incidence of active tuberculosis compared to the general population. Rheumatologists should be aware of both reactivation LTBI and de novo tuberculosis.

## INTRODUCTION

The management of spondyloarthritis (SpA) has been revolutionised in recent years with the advent of biologic disease-modifying antirheumatic drugs (bDMARDs). These drugs, such as tumour necrosis factor-alpha (TNF-alpha) inhibitors, agents targeting the Interleukine-12/23 (IL-12/23) and interleukine-17 (IL-17) pathways, and Janus kinase (JAK) inhibitors have improved therapeutic outcomes and reduced disease progression.^1^

However, it has been shown that patients treated with biologic treatments are at an increased risk of developing infections, including tuberculosis (TB). This risk is particularly high when treated with TNF-alpha inhibitors because TNF alpha plays a critical role in the immune mechanism against *Mycobacterium tuberculosis.*^2,3^ On the other hand, although biologic agents targeting IL-12/23 and IL-17 seem to be associated with a lower risk of developing active TB, long term data is still limited.^4^

Tuberculosis is an endemic disease in Morocco, with an incidence rate of 93 new cases per 100 000 inhabitants annually in 2022, which is higher compared to European countries.^5^ This incidence is due in large part to the reactivation of latent tuberculosis infection (LTBI), which can be caused by several factors, including the use of immunosuppressive drugs.^6^ Therefore, it's important to be vigilant when managing Moroccan patients with inflammatory rheumatic diseases (IRD) who are being treated with biotherapies agents. In 2021, the Moroccan Society of Rheumatology (SMR) established national guidelines for screening and treating tuberculosis in patients with IRD who are receiving biologics.^7^ However, there is still limited data on this significant public health concern in this category of patients.

The primary aim of this study is to determine the incidence of active tuberculosis in patients with SpA who are undergoing biotherapy in the Moroccan biotherapy registry (RBSMR) during a 36-month follow-up period. The secondary objectives are to identify the characteristics of this population and to estimate the rate of latent TB reactivation during follow-up.

## METHODS

### RBSMR study

Our study is based on data from the RBSMR (Registre des Biotherapies de la Société Marocaine de Rhumatologie). This is a multicentre historical-prospective registry of patients over 18-years old who are initiating or ongoing biotherapy for rheumatoid arthritis (RA) or spondyloarthritis (SpA). A total of 419 patients were included (225 RA and 194 SpA) between May 2017 and January 2019 from the ten rheumatology departments of the university hospitals of Morocco. These patients either initiated a bDMARD during this period or were already ongoing biotherapy. Baseline data was collected by clinicians from all medical centres at the time of inclusion. The patients were followed up for a period of three years. Efficacy and tolerance were assessed every six months and whenever patients experience an adverse event or a change in treatment.^8^ This study was designed to assess the incidence of active tuberculosis under biotherapy in patients with SpA, a further study concerning patients with RA is planned.

### Inclusion criteria

We included in this study patients over 18-years old, who fulfilled the ASAS criteria for SpA and who had initiated or were currently undergoing biotherapy.

#### Exclusion criteria

We excluded patients under biotherapy for rheumatoid arthritis and juvenile idiopathic arthritis.

#### Data collection

Details concerning demographic data (age, sex, marital status, educational level), comorbidities, disease characteristics (axial involvement, peripheral involvement, enthesitis, extra-musculoskeletal manifestations, disease duration), treatments history (previous use of biologic therapy, conventional synthetic Disease Modifying Anti Rheumatic Drug (csDMARDs) and corticosteroids) were collected. We also recorded tuberculosis related data including past history of TB infection, LTBI screening before initiating biologic and LTBI prophylaxis.

Definition and incidence of active tuberculosis Active TB was defined as having symptoms consistent with TB and confirmed by positive microbiological evidence of TB (positive acid-fast bacilli smear, TB culture, or polymerase chain reaction). Cases of active TB were recorded from the first day of biotherapy until the date of TB infection, death, or discontinuation of biologic agents. The incidence rate of active TB was calculated per 1000 patient-years of exposure.

#### Definition and screening of LTIB before biotherapy

Moroccan patients with IRD initiating biotherapy particularly TNF inhibitors were systematically referred to pulmonary medicine department for LTBI screening. LTBI was defined as a positive Tuberculin skin tests (TST) with a cut-off greater than 5 mm and/or a positive interferon-gamma release assay (IGRA) after ruling out an active TB infection. Concerning the IGRA test, the commercial technique used in Morocco is the Quantiferon®-TB Gold In-Tube test (QFTGIT) based on the ELISA method. The test was performed in accordance with the manufacturer's instructions.

### Statistical analysis

Statistical analysis was performed using SPSS software, version 13.0. Normally distributed continuous variables were presented as mean ± standard deviation (SD), and asymmetric variables were expressed as median ± interquartile range (IQR defined as 25–75th percentiles). Qualitative data were presented as frequencies (number and percentage). The comparisons between TB and non-TB patients were examined using the T student and Mann-Whitney tests for quantitative variables and using the Chi-squared test or Fischer's exact test for qualitative variables. p values less than 0.05 were considered statistically significant.

### Ethics approval and consent to participate

The protocol for the original RBSMR study was reviewed and approved by local institutional review boards and the national ethic committee: Ethics committee for biomedical research Mohammed V university- RABAT. Faculty of medicine and pharmacy of RABAT. Approval number: 958/09/19 and date: September 11, 2019. Written informed consent for publication was obtained from the patients.

## RESULTS

One hundred and ninety-four patients were included. The demographics and clinical characteristics at the time of inclusion are listed in **Table 1**. 123 were male (63.4%), the mean age was 40.2±13.6 years-old, and the mean disease duration was 11.2±7 years. Biologics used were, in descending order, etanercept (33.3%), adalimumab (30.4%), infliximab (25.3%), golimumab (9.8%), and secukinumab (1.2%). At the time of inclusion, the majority of patients (70.3%) were receiving a first-line biologic agent, 22.1% a second-line, 6.2% a third-line, and 1.4% a fourth-line bDMARD. 6.6% of the patients had a history of treated tuberculosis infection, and none of them were under biologic agent at the time of developing TB infection.

**Table 1.** Demographic, clinical and therapeutic patient characteristics at the inclusion.

		**Total (194)**

**Age, year (mean, SD)** ^1^		40.2±13.6

**Sex, male (n, %)** ^2^		123 (63.4)

**Hypertension** ^2^		11 (5.6)

**Diabetes** ^2^		10 (5.1)

**Active smoking** ^2^		21 (10.8)

**Disease duration (years)** ^1^		11.2±7

**Axial involvement** ^2^		186 (95.9)

**Peripheral involvement** ^2^		134 (69.1)

**Enthesitis** ^2^		115 (59.3)

**HLA B27** ^2^		35 (18)

**Uveitis** ^2^		27 (13.9)

**Psoriasis** ^2^		13 (6.7)

**Inflammatory bowel disease (IBD)** ^2^		20 (10.2)

**Coxitis (hip involvement)** ^2^		79 (40.7)

**Methotrexate** ^2^		52 (26.5)

**bDMARD in inclusion**	**TNF-inhibitors** ^2^	191 (98.8)
	• ETN^2^	64 (33.3)
	• ADA^2^	59 (30.4)
	• IFX^2^	49 (25.3)
	• GOLI^2^	19 (9.8)
	**Anti-IL17** ^2^	3 (1.2)

**Line of bDMARD in inclusion**	**First-line** ^2^	137 (70.3)
	**Second-line** ^2^	43 (22.1)
	**Third-line** ^2^	12 (6.2)
	**Fourth-line** ^2^	2 (1.4)

**Glucocorticoids** ^2^		43(22.2)

**History of previous tuberculosis** ^2^		13 (6.6)

1 Mean and standard deviation,

2 Number and percentage

IBD: inflammatory bowel disease; bDMARD: biologic Disease Modifying Anti Rheumatic Drugs; ETN: etanercept; ADA: adalimumab; IFX: infliximab; GOLI: golimumab.

After 3 years of follow-up, 10 cases of active tuberculosis were recorded with an incidence of 17/1000 patient-years. Characteristics of tuberculosis cases were described in **Table 2** and **3**. The mean age of the patients was 33.1±9.1 years old, and the duration of disease and exposure to biologics were 12.1±7.3 and 7±2.9 years, respectively. Two patients had a history of treated tuberculosis and diabetes, while only one patient received corticosteroid therapy. Tuberculosis was pulmonary in 6 patients while 4 patients have extrapulmonary involvement. All of these patients were on TNF-alpha inhibitors: Adalimumab (n = 5), Infliximab (n = 3), Golimumab (n = 1), Etanercept (n = 1).

**Table 2.** Description of the tuberculosis cases.

	**Age (years)**	**Sex**	**Diabetes**	**Smoking**	**History of treated TB**	**LTBI screening**	**LTBI (+)**	**LTBI Treatment**	**GCs**	**CsDMARDs**	**Number of previous bDMARDs**	Type of biologic^*^	**Exposure duration (years)**	**TB localisation**
1	23	M	No	No	No	TST	Yes	Isoniazid 6 months	No	No	2	ADA	8	Pulmonary
2	52	M	Yes	No	Yes	TST	Yes	Isoniazid 6 months	No	Yes	1	ADA	11	Pulmonary
3	32	M	No	Yes	No	TST and IGRA	No	_	No	Yes	2	ADA	5	Pulmonary
4	37	M	No	No	No	TST and IGRA	No	_	No	No	1	ADA	4	Pulmonary
5	29	F	No	No	Yes	TST and IGRA	No	_	Yes	No	2	GOLI	5	Pulmonary
6	43	M	No	No	No	IGRA and TST	No	_	No	No	1	IFX	11	Extra-pulmonary
7	27	M	No	No	No	IGRA	No	_	No	Yes	2	ADA	5	Extra-pulmonary
8	22	M	No	No	No	IGRA and TST	No	_	No	No	1	ETN	6	Extra-pulmonary
9	31	F	No	No	No	IGRA et TST	No	_	No	Yes	2	IFX	7	Pulmonary
10	36	F	Yes	No	No	IGRA et TST	No	_	No	Yes	2	IFX	5	Extra-pulmonary

M: male; F: Female; TB: Tuberculosis; LTBI: latent tuberculosis infection; GCs: Glucocorticoids; CsDMARDs: Conventional synthetic Disease Modifying Anti Rheumatic Drugs; TST: Tuberculin skin tests. IGRA: Interferon-gamma release assay; ADA: adalimumab; IFX: infliximab; ETN: etanercept; GOLI: golimumab.

* Type of bDMARD at the time of tuberculosis infection.

**Table 3** shows the comparison between patients with active TB and control group. There were no statistically significant differences in age, sex, biologic exposure duration, CsDMARDs, and corticosteroid use, as well as history of treated or latent tuberculosis infection. However, diabetes was significantly more frequent in active TB patients (P = 0.02), as was the prior use of at least two biologic agents (P = 0.03).

**Table 3.** Comparison between the characteristics of tuberculosis and non-tuberculosis patients Tuberculosis patients.

	**TB (10)**	**Non-TB (184)**	**p**
**Age, year (mean, SD)** ^1^	33.1±9.1	40.6±13.8	0.09
**Sex, male (n, %)** ^2^	7 (70%)	116 (63%)	0.65
**Disease duration (years)** ^1^	12.1±7.3	11.1±7	0.69
**Diabetes** ^2^	2 (20%)	8 (4.3%)	0.02
**Smoking** ^2^	1 (10%)	20 (10.9%)	0.93
**Glucocorticoid** ^2^	1 (10%)	43 (23.4%)	0.31
**CsDMARDS** ^2^	5(50%)	102 (55.4%)	0.82
**History of tuberculosis** ^2^	2 (20%)	11 (6%)	0.09
**LTBI** ^2^	2 (20%)	42 (22,8%)	0.62
**Biologic exposure duration (years)** ^2^	7±2.9	5.4±2.4	0.05
**Previous bDMARDs (at least 2)** ^2^	6(60%)	53(28.8%)	0.03

1 Mean and standard deviation,

2 Number and percentage.

TB: tuberculosis; csDMARDs: Conventional synthetic Disease Modifying Anti Rheumatic Drugs; LTBI: latent tuberculosis infection.

**Figure 1.**
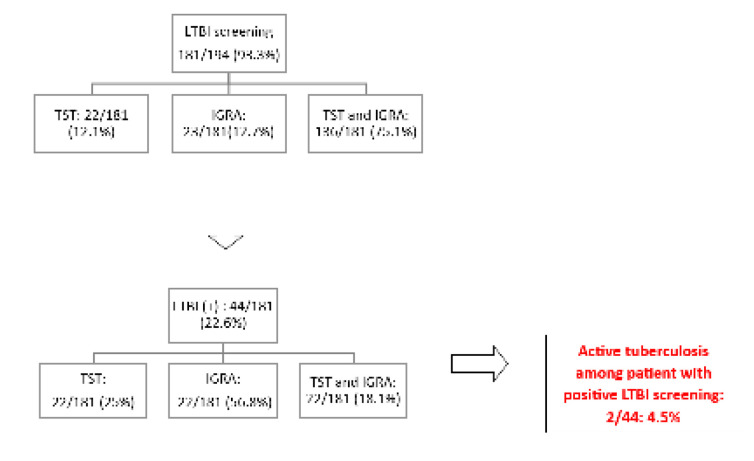
LTBI screening before initiating biotherapy. LTBI: latent tuberculosis infection; TST: Tuberculin skin tests; IGRA: Interferon-gamma release assay.

Screening for latent tuberculosis infection (LTBI) before initiation of biotherapy was conducted in 181 patients. As shown in **Figure 1**, both TST and IGRA were performed in 75.1% of patients, while TST alone was performed in 12.1% and IGRA alone in 12.7%. Of those screened, 22.6% had a positive result and received a chemoprophylaxis. After a 3-year follow-up, only 2 (4.5%) cases of active TB were noted in patients previously treated for LTBI, whereas the other 8 cases had negative screening.

## DISCUSSION

This is the first Moroccan study evaluating prospectively the occurrence of active tuberculosis under biotherapy in patients affected by SpA. 10 cases were recorded during three years of follow-up, with an incidence rate of 17 cases per 1000 patient-year, which is higher comparatively to the incidence in general population in Morrocco. When analysing studies conducted in other countries with a high prevalence of TB, a retrospective Brazilian study conducted in 5853 patients with IRD found an incidence of 2.8 per 1000 patient-years.^9^

Another Brazilian study, which included 221 patients treated with TNF-inhibitors for ax-SpA and psoriatic arthritis, recorded 11 cases of tuberculosis.^10^ An Indian study of 195 patients with RA and SpA found 7 cases of tuberculosis.^11^ Furthermore, data from the French RATIO registry (Research Axed on Tolerance of Biotherapies) showed that the incidence rate of tuberculosis was 116.7 per 100 000 patient-years. This is noteworthy because France has a much lower incidence of TB in the general population.^12^ This finding suggests that the use of biologic agents may increase the risk of active tuberculosis, even in non-endemic countries.

The occurrence of tuberculosis in our immunocompromised patients may be related to several factors. Our study found that diabetes was significantly predominant in the active TB group, which is consistent with literature data.^13^ A systematic review of 13 observational studies found that diabetes increases the risk of TB by three-fold.^14^ In a multicentre cohort study conducted in Morocco on patients under biotherapy, DM was identified as a risk factor for developing active TB.^15^ However, this was a retrospective study conducted on a different population from ours, making it difficult to draw meaningful comparisons with our results. Furthermore, a study conducted in Thailand reported a 42.6% prevalence of DM in patients with tuberculosis.^16^ Our study found that multiple switches between biologics were also more significant in the TB-group. There is no data regarding this association in the literature. However, the Brazilian study mentioned above observed that patients who developed TB had a longer exposure duration to TNF-inhibitors.^10^ This finding may suggest a potential immunocompromising effect induced by biologic exposure.

Other risk factors were identified in the literature, such as consumption of unpasteurized dairy products, smoking, cancer, overcrowding, and malnutrition.^16^ Although we did not find any association between the use of csDMARDs and corticosteroids and tuberculosis in our patients, a study conducted on patients with systemic lupus demonstrated that the cumulative dose of prednisolone was reported as an independent risk factors for developing TB.^17^

Patients receiving biotherapy may develop tuberculosis, either as a new case or as a reactivation of a latent infection.^18^ Therefore, LTBI screening before initiating biotherapy with the two methods: TST and IGRA is a crucial step. The sensitivity and specificity of both tests cannot be accurately measured due to the absence of gold standards for defining LTBI. TST can detect previous infection with *Mycobacterium tuberculosis* or other mycobacteria, as well as previous BCG vaccination which can lead to false-positive results. In contrast, most of studies suggest that IGRA tests are more sensitive and specific. However, false negative results can occur with both tests in patients treated with glucocorticoids and immunosuppressive agents, due to T-cell anergy.^19–21^ In the current stat, international guidelines including those of the Moroccan Society of Rheumatology, recommend that screening for LTBI should be performed by TST and/or IGRA.^7^ These recommendations were not established at the moment of patient inclusions in RBSMR, then, systematic screening for latent tuberculosis was reported in 94% of our patients, and the prevalence of LTBI before initiating bDMARDs was 22.6%.

Assessing the efficacy of chemoprophylaxis in patients with LTBI presents a challenge. Although international recommendations for managing tuberculosis in patients receiving biologic agents have been introduced, several cases of reactivation have been reported. A retrospective study conducted in Greece reported that in 613 patients who received TNF-inhibitors from July 2000 to June 2004, 11 cases of active TB developed. Of these 11 cases, seven occurred in patients who had completed chemoprophylaxis.^22^ In an Italian retrospective series including 500 patients under bDMARDs for psoriatic arthritis, one case of reactivation of LTBI after 2 years of etanercept was reported despite adequate chemoprophylaxis.^23^ In our study, two cases of active tuberculosis were recorded among patients with positive LTBI screening, despite the fact that all LTBI patients received chemoprophylaxis. According to the literature, this condition may be related to several factors, including non-adherence to recommendations, inadequate duration of therapy, and immunosuppressive conditions.^3^ In our series, we were unable to analyse the risk factors due to the small number of cases detected. Additionally, we do not have sufficient data on chemoprophylaxis compliance.

In our study, all patients who developed active tuberculosis were under TNF-inhibitors particularly Adalimumab and Infliximab. This finding is consistent with literature data. It has been demonstrated that TNF-alpha plays a vital role in the host defence mechanism against *Mycobacterium tuberculosis* and in the formation of granuloma that limits the spread of infection. Several studied have recorded an increased incidence of TB in patients with IRD particularly under TNF-inhibitors.^3^ According to a French prospective study, the annual incidence rate of TB was 9.3 per 100 000 for patients receiving etanercept compared to 187.5 per 100 000 for infliximab, and 215.0 per 100 000 for adalimumab.^12^ Similarly, another study found that the incidence rate of TB was highest for adalimumab (144/100 000 person-years), followed by infliximab (136/100 000 person-years), and then etanercept (39/100 000 person-years).^24^

Concerning other biologics agents, active tuberculosis was not reported in a cohort of 12000 patient with psoriasis and spondyloarthritis treated with Secukinumab.^25^ Moreover, there were no instances of active TB development under jak-inhibitors compared to TNF inhibitors in a prospective Korean cohort.^26^ Only 1% of our registry was treated with Secukinumab; therefore, the data was insufficient to draw strong conclusions. Regarding TB localisation, extra-pulmonary involvement was found in 40% of our patients, which is consistent with the results of previous publications. In a Brazilian study, anti-TNF α therapy was identified as a major risk factor for extrapulmonary and disseminated TB forms among patients with RA.^27^

## STRENGTHS AND LIMITATIONS OF THE STUDY

To our knowledge, this is the first Moroccan prospective study assessing tuberculosis incidence in patients undergoing biologics agents. However, it presents some limitations. First, some information was missing due to the fact that we used data from a national registry. Second, there were not recommendations established by Moroccan Society of Rheumatology concerning systematic LTBI screening before the initiation of biologic therapy at the moment of inclusion. Therefore, only 13/194 were not screened for LTBI. Third, the median time from previous active TB treatment to the start of follow-up and some risk factors such as occupation and household contact among patients with active TB were not recorded in our registry. Fourth, only patients with spondyloarthritis were included. And finally, the limited number of tuberculosis cases was a methodological limitation to conduct multivariate regression analysis to assess related risk factors.

In conclusion, our study suggests that patients undergoing biotherapy have a higher incidence of active tuberculosis compared to the general population. The group with tuberculosis had a more significant frequency of DM, corticosteroid use, and required at least two TNF-inhibitors. Additionally, the study recorded cases of both new tuberculosis cases and reactivation of latent infections despite chemoprophylaxis. Therefore, it is recommended that more rigorous screening strategies be implemented for this patient category particularly in endemic countries. Screening for LTBI should be included, along with an assessment of various immunocompromising factors.

## AUTHOR CONTRIBUTIONS

Salma Zemrani drafted the manuscript and reviewed the literature. Bouchra Amine and Imane Elbinoune participated in article writing and reviewed critically the manuscript. Samira Rostom and Rachid Bahiri reviewed critically the manuscript. The scientific committee of the RBSMR study has reviewed and approved the final manuscript.

## CONFLICTS OF INTEREST

All authors do not report any conflict of interest.

## FUNDING

All authors report no external funding for this work.

## ETHICS APPROVAL AND WRITTEN INFORMED CONSENTS STATEMENT

The protocol for the original RBSMR study was reviewed and approved by local institutional review boards and the national ethic committee: Ethics committee for Biomedical Research Mohammed V University-RABAT, Faculty of Medicine and Pharmacy of RABAT. Approval number: 958/09/19 and date: September 11, 2019. Written informed consent for publication was obtained from the patients.
